# Risk of pre-term births and major birth defects resulting from paternal intake of COVID-19 medications prior to conception

**DOI:** 10.1186/s13104-020-05358-x

**Published:** 2020-11-07

**Authors:** Silvia Rizzi, Maarten J. Wensink, Rune Lindahl-Jacobsen, Lu Tian, Ying Lu, Michael L. Eisenberg

**Affiliations:** 1 Department of Epidemiology, Biostatistics and Biodemography, University of Southern Denmark, Odense, Denmark; 2 Interdisciplinary Centre on Population Dynamics, University of Southern Denmark, Odense, Denmark; 3 Department of Biomedical Data Science, Stanford University School of Medicine, Stanford, USA; 4 Male Reproductive Medicine and Surgery, Department of Urology and Obstetrics & Gynecology, Stanford University School of Medicine, 300 Pasteur Drive, S-287, Stanford, CA USA

**Keywords:** COVID-19, Paternal reproductive safety, Pharmacological treatment, Pre-term birth, Congenital malformation

## Abstract

**Objective:**

With the ongoing COVID-19 pandemic, large numbers of people will receive one of the several medications proposed to treat COVID-19, including patients of reproductive age. Given that some medications have shown adverse effects on sperm quality, there might be a transgenerational concern. We aim at examining the association between drugs proposed to treat COVID-19 when taken by the father around conception and any pre-term birth or major birth defects in offspring in a nation-wide cohort study using Danish registry data. Offspring whose father filled at least one prescription of the following medications in the 3 months preceding conception were considered exposed: chloroquine, hydroxychloroquine, losartan, azithromycin, naproxen, dexamethasone and prednisone.

**Results:**

For azithromycin and naproxen, large numbers of offspring were exposed (> 1800 offspring), and we found no association with adverse birth outcomes. For chloroquine, losartan and dexamethasone, exposure was intermediate (~ 900 offspring), and there was no statistically significant association with birth defects. For hydroxychloroquine and prednisone, exposure was limited (< 300 offspring). Our evidence suggests that azithromycin and naproxen are safe with respect to pre-term birth and birth defects. For the other drugs investigated larger exposures are needed for conclusive statements.

## Introduction

To find an effective treatment for COVID-19 several existing drugs have been assessed and approved for clinical trials through accelerated procedures worldwide [1, 2]. These drugs meant to treat non COVID-19 specific conditions are now given massively to patients affected by COVID-19.

Evidence suggests that males are more severely affected by the ongoing pandemic irrespective of age [3] and therefore are exposed to more proposed treatments. Importantly, while most COVID-19 patients who require treatment are beyond reproductive age, the epidemiological profile of this pandemic suggests that patients also include men and women in their respective reproductive ages. The age distribution of patients has varied across countries and over time. As for end of summer and beginning of autumn 2020, an increase of young adults affected is observed.

We aimed to investigate the safety of COVID-19 drugs taken by fathers prior to conception to assess for adverse birth outcomes. A few recent studies have shown adverse effects of drugs, including chloroquine, on gametes [4–6], raising a potential transgenerational concern. In the present cross-sectional study we analyze the association between the use of COVID-19 medications used by fathers three months before conception and pre-term birth and congenital malformations, using the Danish National Birth, Patient and Prescription Registries [7–9] from 1997 to 2017.

## Main text

### Methods

#### Population

The Danish Birth Registry (MFR) [7] records information on all children born in Denmark including the father (if known), the mother, and basic vital information, such as Apgar score, birth weight, birth length, and gestational age. We restricted the analysis to live born singletons for the time frame 1997–2016 (1,201,131 births).

The Danish National Patient Registry (DNPR) [8] provides administrative and clinical data of all patients discharged from Danish hospitals and outpatient clinics. One primary and optional secondary diagnoses are recorded according to the International Classification of Diseases for each visit. Using the CPR number of the Danish Birth Registry, data of the Danish National Patient Registry were linked at the patient level for all children. Allowing 1 year after birth for follow-up, we identified all newborns who were found to have at least one major birth defect as per EUROCAT Guide 1.4 [10]. Hence, the last cohort of live born singletons of 2016 is followed up to 2017 in the National Patient Registry.

The Danish Birth Registry can be linked to the Danish National Prescription Registry (LMDB) [9] through the CPR number of the father. This registry includes all medications prescribed by a medical doctor. We examined the medications that are being trialed as treatments for COVID-19. The list of medications was retrieved from the Danish Medicines Agency [11]. We included those drugs with adequate usage to have a reasonable power: chloroquine, hydroxychloroquine, losartan, azithromycin, naproxen, dexamethasone and prednisone. We examined prescriptions filled in the three months prior to conception based on a spermatogenesis cycle. Using the CPR number of the father, also the birth day, income, and the highest educational attainment of the father was retrieved from the population and education registries held at Statistics Denmark.

#### Outcome

Our two primary outcomes were odds of pre-term birth (birth at less than 37 weeks of gestation) and of having at least one major birth defect in offspring (as per EUROCAT Guide 1.4 [10]).

#### Statistical analysis

All analyses were carried out at the secure server of Statistics Denmark in R (version 3.6.1) [12]. Conception date was calculated as the birth date minus gestation age as held in the registry. Infants born to fathers who had at least one prescription within three months before conception were considered exposed. Education of the father was simplified to three levels: low, middle and high. Smoking status of the mother during pregnancy was reduced to three levels: no smoker, past smoker, and current smoker. To handle missing data (< 5%) we created five imputed datasets using multiple imputation by chained equations with the R package *mice* (version 3.6.1) using predictive mean matching for the numeric variables and polytomous regression for the categorical variables. We performed logistic regression on each of the medications separately, adjusting for birth year, the age of both parents, smoking status of the mother, and education and income of the father. In addition, the regressions of major birth defects were adjusted for gestation age. We also performed logistic regression with all medications as independent variables simultaneously. The statistical significance threshold for all tests is set at two-side 0.05 level.

### Results

The average age of fathers was 32.3 years, while mothers were on average 29.7 years old. Of the mothers, 84.1% did not smoke at conception, 2.6% quit during pregnancy and 13.3% smoked during pregnancy. Of the fathers, 79.9% had at least upper secondary education, of which 11.8% had at least a masters degree.

Medications taken by a large number of fathers (> 100 fathers) were chloroquine, hydroxychloroquine, losartan, azithromycin, naproxen, dexamethasone, and prednisone.

The overall birth defect rate was 3.2%, while 7.4% of all births were preterm.

None of the investigated drugs taken by men up to three months before conception was statistically associated with pre-term birth or congenital malformations at 5% significant level (Table 1). Including all the medications in the same regression model yielded identical results.

**Table 1:** Paternal medication taken around conception and births (singleton and alive), full term, pre-term, and with birth defects in Denmark for birth cohort 1997–2016: summary of data and results of logistic regression model

Medication	ATC code	Births	Full term	Pre-term (%)	Birth defect (%)	Pre-term vs. full term			Birth defect vs. not		
		1,201,131	1,087,841	86886 (7.40%)	38881 (3.20%)	OR (95% CI)	p-value	Detectable OR^b^	OR (95% CI)	p-value	Detectable OR^b^
Azithromycin	J01FA10	7278	6721	557 (7.7%)	235 (3.20%)	1.01 (0.93–1.10)	0.77	1.13	0.98 (0.86–1.12)	0.76	1.19
Naproxen	M01AE02	1895	1749	146 (7.7%)	63 (3.30%)	1.01 (0.85–1.20)	0.9	1.26	1.05 (0.82–1.35)	0.69	1.38
Losartan	C09CA01	972	865	69 (7.40%)	43 (4.60%)	1.01 (0.79–1.29)	0.96	1.37	1.3 (0.96–1.77)	0.094	1.55
Dexamethasone	H02AB02 S01BA01 S03BA01	907	838	69 (7.60%)	32 (3.50%)	1.05 (0.82–1.34)	0.72	1.38	1.11 (0.78–1.58)	0.57	1.57
Chloroquine	P01BA01	849	789	60 (7.10%)	30 (3.50%)	0.98 (0.75–1.28)	0.89	1.39	1.32 (0.91–1.90)	0.14	1.59
Prednisone	H02AB07	289	262	27 (9.30%)	9 (3.10%)	1.26 (0.85–1.87)	0.25	1.70	1 (0.51–1.94)	0.99	2.06
Hydroxychloroquine	P01BA02	101	> 95^a^	< 6^a^	< 6^a^	0.38 (0.12–1.21)	0.1	2.22	1.22 (0.45–3.32)	0.7	3.00

Exposure: male preconception three months

Births, full term, pre-term, and birth defect columns contain counts and percentage in brackets

Logistic model adjusted for age of mother, age of father, birth year, education and income of the father, smoking status of the mother. Birth defects additionally adjusted for gestation age

^a^Privacy regulations of Statistics Denmark do not allow publication of numbers smaller than 6

^b^Detectable OR is the alternative hypothesis that can be detected with 80% power given the observed sample size and 2-sided 5% type I error rate without any adjustment of covariates

For hydroxychloroquine and prednisone, exposure was limited (< 300 offspring). For azithromycin and naproxen, large numbers of offspring were exposed (> 1800), and we found no association with adverse birth outcomes with 95% confidence interval of odds ratios given in Table 1. For chloroquine, losartan and dexamethasone, exposure was intermediate (~ 900 offspring), while there was a no statistically significant association with birth defects. Because of the low exposure, even using the Danish population data, the detectable odds ratio for 80% power is still very high for the rarely exposed medications.

### Discussion and conclusion

Using a nationwide cross-sectional study with data over 20 years in Denmark, we found no statistical evidence of association between the COVID-19 drugs under study, taken by men up to three months before conception, and pre-term birth or major birth defects in offspring. Evidence of drugs safety before paternal conception is limited. Our findings suggest that azithromycin and naproxen can be considered safe with respect to pre-term birth and major birth defects. For chloroquine, losartan and dexamethasone, our study was more constrained in size, while for hydroxychloroquine and prednisone we would be able only to find very large effects. While reassuring, given the limitations of the current report, continued surveillance of parental exposure to experimental COVID-19 treatments remains prudent.

## Limitations

The prescription registry does not include prescription diagnosis. It was therefore not possible to separate the effect of the medication from the effect of the disease for which the medication was given. This study will therefore tend to overestimate, but not underestimate any effect. This might (partly) explain odds ratios larger than one for chloroquine and losartan. In addition, medications that can be obtained over the counter (e.g. naproxen) may be misclassified biasing results toward the null.

## Data Availability

All data are held at the secured server of Statistics Denmark and can be processed only there due to privacy regulations.

## Abbreviations

MFR: Danish Birth Registry
DNPR: Danish National Patient Registry
LMDB: Danish National Prescription Registry
CPR: Central Person Register
EUROCAT: European Registry of Congenital Anomalies and Twins
